# Electrospun Nanofibers Loaded with Marigold Extract Based on PVP/HPβCD and PCL/PVP Scaffolds for Wound Healing Applications

**DOI:** 10.3390/ma17081736

**Published:** 2024-04-10

**Authors:** Magdalena Paczkowska-Walendowska, Natalia Rosiak, Tomasz Plech, Tomasz M. Karpiński, Andrzej Miklaszewski, Katarzyna Witkowska, Maciej Jaskólski, Cansu Erdem, Judyta Cielecka-Piontek

**Affiliations:** 1 Department of Pharmacognosy and Biomaterials, Poznan University of Medical Sciences, Rokietnicka 3, 60-806 Poznan, Poland; mpaczkowska@ump.edu.pl (M.P.-W.); nrosiak@ump.edu.pl (N.R.); witk.katarzyna@gmail.com (K.W.); jaskolski.mj@gmail.com (M.J.); cansueerdem@gmail.com (C.E.); 2 Department of Pharmacology, Medical University of Lublin, Radziwillowska 11, 20-080 Lublin, Poland; tomasz.plech@umlub.pl; 3 Faculty of Medicine, Mazovian Academy in Płock, 09-402 Płock, Poland; 4 Department of Medical Microbiology, Medical Faculty, Poznan University of Medical Sciences, Rokietnicka 10, 60-806 Poznan, Poland; tkarpin@ump.edu.pl; 5 Faculty of Materials Engineering and Technical Physics, Institute of Materials Science and Engineering, Poznan University of Technology, 60-965 Poznan, Poland; andrzej.miklaszewski@put.poznan.pl; 6 Department Pharmaceutical Chemistry, Ege Üniversitesi, 35040 İzmir, Turkey

**Keywords:** marigold flower, *Calendulae flos*, chlorogenic acid, nanofibers, electrospinning, wound healing

## Abstract

Marigold flower is a traditionally used plant material topically applied on the skin due to its anti-inflammatory properties and antibacterial activity. This potential of action justifies the implementation of marigold extract in nanofiber scaffolds based on poly-vinylpyrrolidone/hydroxypropyl-β-cyclodextrin (PVP/HPβCD) and polycaprolactone/polyvinylpyrrolidone (PCL/PVP) obtained by electrospinning for wound treatment. Using SEM, the morphology of electrospun scaffolds showed a fiber diameter in the range of 298–527 nm, with a uniform and bead-free appearance. ATR-FTIR spectroscopy confirmed the presence of marigold extracts in nanofibrous scaffolds. The composition of the nanofibers can control the release; in the case of PVP/HPβCD, immediate release of 80% of chlorogenic acid (an analytical and functional marker for marigold extract) was achieved within 30 min, while in the case of PCL/PVP, the controlled release was achieved within 24 h (70% of chlorogenic acid). All systems showed weak antibacterial activity against skin and wound-infecting bacteria *Staphylococcus aureus* (MIC 100 mg/mL), and *Pseudomonas aeruginosa* (MIC 200 mg/mL) and yeasts *Candida albicans* (MIC 100 mg/mL). Analysis of the effect of different scaffold compositions of the obtained electrofibers showed that those based on PCL/PVP had better wound healing potential. The scratch was closed after 36 h, compared to the 48 h required for PVP/HPβCD. Overall, the study shows that scaffolds of PCL/PVP nanofibers loaded with classic marigold extract have the best potential as wound dressing materials because of their ability to selectively modulate inflammation (via inhibition of hyaluronidase enzyme) and supportive antimicrobial properties, thereby aiding in the early stages of wound healing and repair.

## 1. Introduction

Over the past few decades, improving and planning for the management chronic wounds has become increasingly important to prolong life and improve human quality of life [1]. Numerous developing technologies are being explored because more complicated and biomimetic tactics are needed [2]. Many of the approaches used are based on polymer scaffolds containing biodegradable polymers, which, depending on their composition and/or structure, offer protection, moisture retention, and therapeutic effects [3]. The development of biopolymeric scaffolds involves various procedures, including conventional casting, hydrogel production, and extracellular matrix decellularization, in addition to innovative electrospinning and 3D bioprinting methods [4].

As an alternative to conventional fabrication techniques, electrospinning has gained popularity because it is an easy-to-use procedure that allows for one to modify the fabrication parameters (such as the nozzle diameter, flowrate, and voltage of the electric fields) to control the porosity and/or morphology of nanofibers [5]. Specifically, the mechanical qualities, high porosity, tunable surface area-to-volume ratio, strong biocompatibility, and good porosity make electrospun nanofibers an attractive choice for wound dressing [6,7], and the combination of producing nanofibers containing plant extracts is becoming an increasingly popular center of interest for scientists [8,9].

Numerous plant raw materials have the potential to heal wounds; the most frequently used raw materials include *Centella asiatica* [9], *Aloe barbadensis* [10], and *Calendula officinalis* [11]. Inflammations of the skin, small cuts, and the mouth or throat have long been treated with alcoholic and oil extracts of *Calendulae flos*, also known as marigold flowers, according to a report from the European Medicines Agency (EMA) [12]. Triterpenes, carotenoids, polyphenols, and other specific categories of secondary metabolites are linked to the anti-inflammatory properties of plant raw materials [12]. Numerous in vitro and in vivo studies have demonstrated the beneficial effects of *Calendulae flos* extracts on wound healing. These studies have shown that the extracts stimulate angiogenesis, decrease collagen degradation, and increase the proliferation and migration of human fibroblasts and keratinocytes [13,14]. Apart from its recommended use in wound care, marigold extract possesses various pharmacological properties, such as anti-inflammatory and antioxidant properties, as well as antibacterial, antifungal, and antiviral properties against a range of pathogens, including *Bacillus subtilis* and *Staphylococcus aureus* [15,16].

To date, several attempts have been made to create innovative wound delivery systems containing marigold extract, also including nanofibers. Chitosan- [11], polyvinyl alcohol- [17], and polyacrylamide-based [18] hydrogels have been tested. Calendula extract loaded in chitosan/polyethylene oxide nanofibrous scaffolds (CS/PEO) have shown the potential to promote collagen synthesis, tissue remodeling, and re-epithelization during wound healing [19]. Moreover, polycaprolactone (PCL), due to its unique structural characteristics, biocompatibility, and slow biodegradation qualities, has produced a promising substrate for various applications, including wound healing nanofibers [20]. PCL/gelatin and PCL/gum arabic nanocomposite scaffolds were found to be appropriate for fibroblast cell proliferation [21,22]. Nanofibers containing polyvinyl alcohol (PVA)/sodium alginate (SAlg) were characterized by proper adhesion to the site of inflammation and favorable release kinetics of active compounds. First, active compounds were released immediately; then, the release profile ensured constant concentrations of active compounds [22]. While the above data provide evidence for the use of both calendula extract and nanofiber structures for wound treatment, they do not indicate the pharmaceutical aspects of such innovative dressings. Therefore, the aim of our work was, apart from the morphological and structural assessment (PVP is designed to store large amounts of water without losing mechanical integrity, and PCL is designed to provide flexibility and biocompatibility), for the first time, to compare the influence of the nanofibrous mat matrix on the release of active compounds and the assessment of biological activity (HPβCD, as the most water-soluble cyclodextrin, is intended to improve the solubility of the extract’s active compounds). For this purpose, two marigold extracts and two different compositions of nanofibers containing substances that dissolve quickly in water like polyvinylpyrrolidone/hydroxypropyl-β-cyclodextrin (PVP/HPβCD) and those with a prolonged dissolution time like polycaprolactone (PCL) were prepared.

The above justifications for using marigold extracts in the form of nanofibers obtained by electrospinning are important; therefore, it is worth expanding the area of research concerning the development of dressings dedicated to wound treatment. In our work, we developed dressings based on scaffolds containing PVP/HPβCD and PCL/PVP. Considering the functional benefits of combining selected biopolymer systems and an extract from the medicinal raw material marigold flower, our work aimed to obtain and fully characterize the identity of the obtained dressings and assess their functionality.

## 2. Materials and Methods

### 2.1. Plant Materials

The flowers of *Calendula officinalis* L. were purchased from the “Kawon-Hurt”, Gostyń, Poland (Lot No. 130.2022).

### 2.2. Chemicals

Chlorogenic acid (Phyproof^®^ Reference Substance) and narcissin = isorhamnetin 3-rutinoside (Phyproof^®^ Reference Substance) were obtained from Sigma-Aldrich (Poznan, Poland). Excipients from Sigma-Aldrich (Poznan, Poland) included polyvinylpyrrolidone K30, (2-hydroxypropyl)-β-cyclodextrin (average Mw ~1460), and polycaprolactone. Sigma-Aldrich (Poznan, Poland) provided reagents for dissolution tests (phosphate buffer) and activity testing (2,2-Diphenyl-1-picrylhydrazyl (DPPH), sodium chloride, bovine serum, hexadecyltrimethylammonium bromide (CTAB), and hyaluronic acid (HA)). Mueller–Hinton agar was obtained from Graso Biotech (Starogard Gdański, Poland). HPLC-grade acetonitrile and water were obtained from Merck (Darmstadt, Germany). High-quality pure water and ultrahigh-quality pure water were prepared using a Direct-Q 3 UV Merck Millipore purification system.

### 2.3. Preparation and Characterization of Calendulae flos Lyophilized Extracts, and Investigation of Biological Activity

Two types of extracts were prepared, (1) using the classical extraction method (CF-CE) and (2) ultrasonic-assisted extraction (CF-UAE). In brief, CF-CE was prepared as follows: three times, 300 g of dried plant material was extracted using ethanol/water (7:3), for 30 min at 95 °C in a water bath each time. The obtained extracts were concentrated under vacuum, and then lyophilized (CHRIST 1-4 LSC, Osterode am Harz, Germany) [11]. CF-UAE was prepared as follows: using an ultrasonic bath, 300 g of dried plant material was extracted 3 times for 30 min at 70 °C. The extracts were combined, concentrated, and lyophilized. The freeze-drying parameters were the same for both extracts and included a condensation temperature set at −48 °C under reduced pressure (1.030 mbar) for 48 h.

The presence and concentration of active substances in the freeze-dried *Calendulae flos* extract were determined using a previously developed, validated HPLC method [11], while the total content of phenolic components was determined spectrophotometrically [23]. Antioxidant action of freeze-dried *Calendulae flos* extract was studied by using the DPPH method [23], and anti-inflammatory activity was expressed as inhibition of hyaluronidase enzyme activity.

The assay that was used to measure the antioxidant activity involved the use of 2,2-Diphenyl-1-picrylhydrazyl (DPPH). Using a turbidimetric technique, the hyaluronidase inhibition approach was ascertained. Both methods have already been described [23].

### 2.4. Electrospun Nanofiber Preparation

Four different types of nanofibers were prepared using NS + NanoSpinner Plus Electrospinning Equipment (Inovenso Ltd., Istanbul, Turkey) (Table 1). Table 1 states that all components were dissolved in the designated solvent for two hours. The solution was then put into a syringe and electrospun at 27 kV voltage, 2 mL/min flow rate, and 12 cm distance. Aluminum foil-wrapped rotary collectors were used to gather the nanofibers.

### 2.5. Identification of the Electrospun Nanofibers

#### 2.5.1. Scanning Electron Microscopy (SEM)

The surface morphology of the nanofiber was observed using SEM. The nanofibers were examined using a Quanta 250 FEG (FEI, Waltham, MA, USA) scanning electron microscope () following gold–palladium sputter coating.

#### 2.5.2. X-ray Diffraction (XRPD)

Using a Bragg–Brentano reflection mode configuration with 45 kV and 40 mA settings, an X-ray diffraction (XRD) apparatus with a copper anode (CuK—1.54 Å) was used to study the sample structure. The apparatus was purchased from Panalytic Epicurean in Almelo, The Netherlands. The measurement parameters were set consistently between 3 and 60°, with a step of 45 s between each degree.

#### 2.5.3. Fourier Transform Infrared Spectroscopy with Attenuated Total Reflectance (ATR-FTIR) and DFT Study

The ATR-FTIR spectra were acquired using an IRTracer-100 (Shimadzu, Kyoto, Japan) spectrophotometer, covering a range from 400 to 4000 cm^−1^ in absorbance mode. The spectrometer settings included a resolution of 4 cm^−1^, 400 scans, and Happ–Genzel apodization. LabSolutions IR software (version 1.86 SP2, Shimadzu, Kyoto, Japan) was employed to compute the second derivative of CF-CE, CF-UAE, narcissin, and chlorogenic acid spectra using the Savitzky–Golay numerical algorithm, with a smoothing parameter set to 11 points. The derivative spectrum facilitated the identification of peak positions in the original spectrum and the separation of closely located or shoulder peaks. The minima of the second derivative corresponded to the extremes of the original ATR-FTIR spectrum. Origin 2021b (OriginLab Corporation, Northampton, MA, USA) was utilized for the analysis of the collected data.

The DFT spectra of narcissin (PubChem CID 5481663) and chlorogenic acid (PubChem CID 1794427) (website: https://pubchem.ncbi.nlm.nih.gov/, accessed on 8 February 2024) were obtained using GaussView software (Wallingford, CT, USA, Version E01), and the normal modes were inspected visually. The molecular geometries of narcissin and chlorogenic acid were optimized using the density functional theory (DFT) method with Becke’s three-parameter hybrid functional (B3LYP) implemented with the standard 6–311G(d,p) as a basis set.

### 2.6. Studies of Electrospun Nanofiber’s Functionality

#### 2.6.1. Release of Active Components

Electrospun nanofibers were subjected to dissolve experiments using Agilent 708-DS dissolving equipment (Santa Clara, CA, USA). A typical basket method was used, with 50 rpm and stirring at 37 ± 0.5 °C. Nanofibers were added to 300 mL of phosphate buffer (pH 5.5), which mimics the pH of skin. At regular intervals, liquid samples were collected, and the same volume of temperature-stabilized medium was swapped out. A nylon membrane filter with a mesh size of 0.45 µm was used to filter the samples. Using the previously mentioned HPLC procedure, the amounts of chlorogenic acid in the filtrated acceptor solutions were ascertained. The resulting active compound release patterns were fitted to the Higuchi, Korsmeyer–Peppas, zero-order, and first-order models in order to examine the release kinetics [24].

#### 2.6.2. Microbiological Activity

Nanofibers N1–N4 were dissolved in pure water, obtaining stock solutions at a concentration of 200 mg/mL. A series of dilutions in the concentration range 25–200 mg/mL were prepared. Chlorogenic acid was dissolved in water, and dilutions ranging from 0.156 to 10 mg/mL were employed. In this study, clinical strains of *Staphylococcus aureus* and *Pseudomonas aeruginosa* bacteria and *Candida albicans* yeast were used. The microbial growth inhibitory potential of the tested nanofibers was determined by using the agar disc diffusion method as described in our previous publication [25]. In brief, the inoculums were adjusted to obtain a final concentration of 10^5^ CFU/mL for bacteria and 10^4^ CFU/mL for fungi. The pathogens were transferred on Mueller–Hinton agar (Graso Biotech, Starogard Gdański, Poland), and 20 µL of each nanofiber or chlorogenic acid dilution was transferred onto sterile filter papers (6 mm diameter). Plates were incubated at 37 °C for 24 h. The presence of a zone of growth inhibition indicated the antimicrobial inhibitory activity (MIC) at a particular concentration of the product.

#### 2.6.3. Wound Healing Assay

Wound healing properties of nanofibers were examined on Hs27 cells using a scratch assay. The American Type Culture Collection (Manassas, VA, USA) provided human skin fibroblasts Hs27 (CRL-1634), which were cultivated in DMEM/high glucose supplemented with 10% FBS, penicillin (100 U/mL), and streptomycin (100 µg/mL). Before the experiment, the Hs27 cells were detached using trypsin/EDTA and, subsequently, seeded on 6-well culture plates (Corning Inc., Corning, NY, USA) at a density of 1 × 10^5^ cells/mL. Next, using a sterile pipette tip, a vertical linear scratch was made in the monolayer when the cell confluence reached roughly 90%. After three rounds of phosphate-buffered saline (PBS) washing to remove any remaining cell debris, new media containing either nanofibers or 2% FBS (control group) was added to the corresponding wells. Following that, pictures of the scratch were captured using an Olympus CKX53 microscope equipped with an XM10 digital camera (Olympus, Warsaw, Poland) at 0 h, 24 h, 36 h, and 48 h. At least two people carried out the experiments. At the start of the trial (0 h), 100% of the scratch area was taken into consideration. NIH ImageJ software (Bethesda, Rockville, MD, USA) (https://imagej.net/nih-image/ access date: 1 September 2023) was used to quantify the open wound area. The following formula was used to determine the wound closure percentage:

$$Wound\;closure\left(\%\right)=\frac{open\;wound\;area\;at\;0\;h-open\;wound\;area\;at\;24/36/48\;h}{open\;wound\;area\;at\;0\;h}\times 100\%$$

### 2.7. Statistical Analysis

Statistica 13.3 was used to conduct the statistical analysis. To check if the data were normal, the Shapiro–Wilk test was performed. The variances between the mean values were investigated using the ANOVA test and Tukey’s post-hoc range test for multiple comparisons. Differences between groups were considered significant at *p* < 0.05. PQStat Software version 1.8.4.142 (2022) was used to evaluate correlations through principal component analysis (PCA).

## 3. Results and Discussion

The first stage of the research was the preparation of marigold flower extract. Based on previous work, the extract was prepared using the classical method, i.e., heating the plant material under a reflux condenser, according to European Pharmacopoeia (Ph. Eur.) 9th Edition, *Calendulae flos* monograph (CF-CE) [11,26], which was an attempt to compare it to the extract prepared using the ultrasonic-assisted extraction method (CF-UAE). The influence of the type of extraction on the content of active compounds expressed as total phenolic content (TPC), chlorogenic acid and narcissin contents measured by validated HPLC method (Table S1, Supplementary Material), and the antioxidant and anti-inflammatory activity of the obtained extracts were assessed (Table 2).

A slightly higher content of active compounds was observed in the case of CF-UAE, as well as increased antioxidant and anti-inflammatory activity. Still, the obtained differences were not statistically significant. Due to the comparable properties of both obtained extracts, it was decided to continue work with the CF-CE and CF-UAE extracts. At this stage, none of them were rejected for further testing.

In this work’s second part, the method of producing electrospun nanofibers loaded with marigold extracts, both CF-CE and CF-UAE, was developed. The aim was to assess the polymer’s impact on the synthesized nanofibers’ pharmaceutical properties. A combination of polyvinylpyrrolidone (PVP) as a substance highly soluble in water and hydroxypropyl-β-cyclodextrin (HPβCD) as a solubilizer was used. This combination was intended to improve the solubility properties of the active compounds in the extract loaded in nanofibers N1 and N2 (Table 1) [27,28]. The second option was to combine polycaprolactone (PCL), a substance known from biomedical engineering and with frankly described properties in wound healing [29], and PVP in nanofibers N3 and N4 (Table 1). Based on preliminary tests, the appropriate concentration of polymers and extracts and the best solvents were selected to prepare the mixture for electrospinning. As a result, N1–N4 nanofibers were obtained, and the process was problem-free and repeatable.

**Figure 1:**
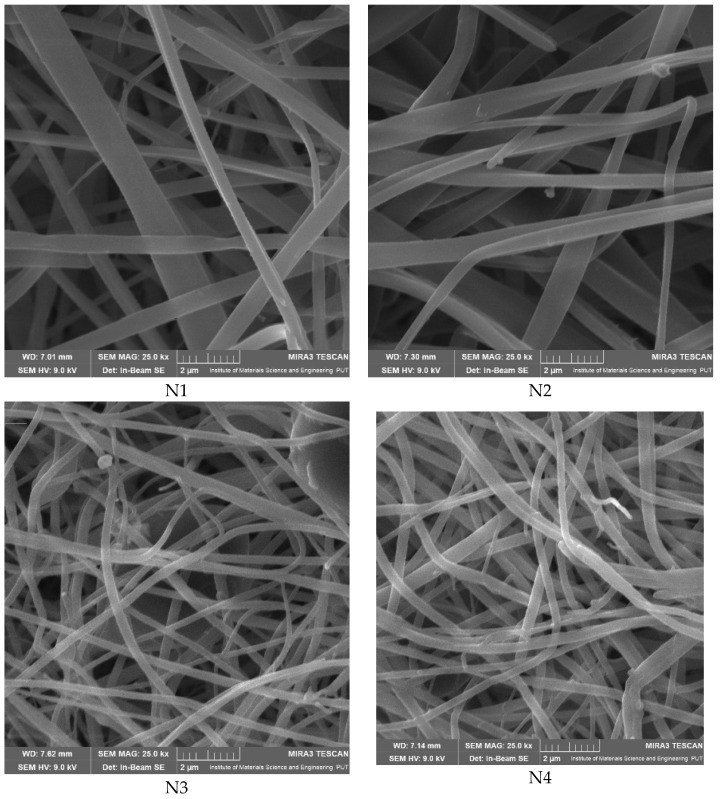
SEM imagines of nanofibers N1–N4.

Then, the formation of nanofibers and the morphology of the electrospun nanofibers N1–N4 were examined using a scanning electron microscope (SEM) (Figure 1). The SEM analysis of nanofibers N1–N4 indicated the formation of smooth, beadless, and uniform nanofibers mats, made up of interconnected, randomly oriented fibers in a three-dimensional, highly porous structure. The analysis of the PCL/PVP nanofiber (N3–N4) diameter distribution, as depicted in Table 3, estimated that the mean fiber diameter was in the range 298–392 nm. It seems that the addition of HPβCD to PVP increased the diameter distribution almost two times (the average diameter of nanofibers N1–N2 was in the range 521–527 nm). Additionally, it is worth noting that PCL/PVP nanofibers (N3 and N4) are characterized by higher uniformity of fiber sizes, and the diameter of fibers decreased with the addition of PCL. Poor spinnability of PVP resulting in large fiber diameter has already been observed, and the addition of PCL increases the solution spinnability, leading to finer fibers [30,31]. Varsei et al. concluded that the PVP concentration is the dominant factor (compared to the PCL concentration and other electrospinning parameters) in the morphology of the nanofibers [32]. This also confirms the results obtained in this work. No significant influence of the type of extract used, CF-CE or CF-UAE, was observed. So, the type of polymer matrix used influences the size of the nanofibers and their uniformity [31].

**Figure 2:**
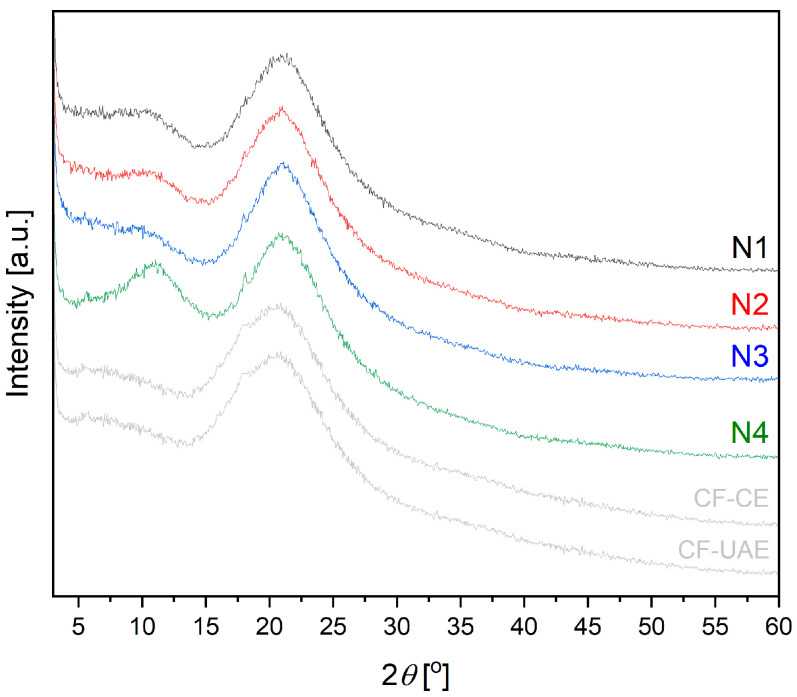
XRPD diffractograms of lyophilized extracts CF-CE and CF-UAE, and nanofibers N1–N4.

X-ray diffraction (XRPD) measurement was carried out to investigate the structural nature of the nanofibers (Figure 2). Marigold lyophilized extract diffractograms showed no crystalline planes. Freeze-drying is a well-known method to produce amorphous solid dispersions with various substances [33]. The raw PVP exhibits two broad peaks at around 11° and 21°, which are referred to the amorphous nature of PVP [34]. The raw PCL is a semi-crystalline polymer with high intensity peaks at 21.25° and 23.7° [35]. The extracts form ultrafine amorphous particles in PVP matrices within nanofibers N1 and N2. With the increase in PVP content in the case of nanofibers N3 and N4, the relative intensity of the PCL hump diminishes while its broadness grows, indicating the success of mixed spinning of PCL and PVP. Only a very low intensity peak at 20° remains visible in nanofibers N3–N4, which means a higher degree of amorphousness of the PVP/HPβCD systems (nanofibers N1–N2). As a result of the complexation between extracts and polymers, no peaks were observed, indicating that the extracts were completely dissociated in polymer matrices. No appearance of a diffraction peak indicates the amorphous nature of nanofibers N1–N4.

**Figure 3:**
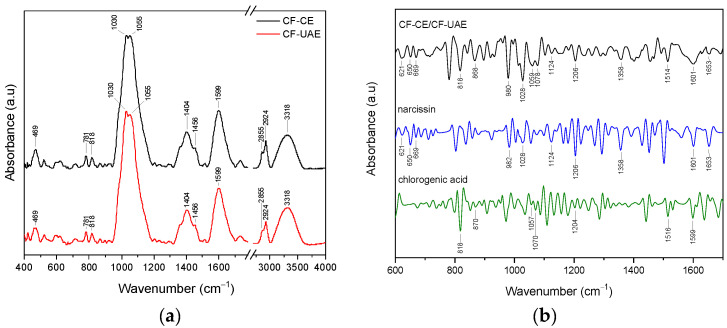
ATR-FTIR analysis of CF-CE (black line), CF-UAE (red line), range 400–4000 cm^−1^ (**a**), and second derivative infrared spectra (by the Savitzky–Golay polynomial fitting method, 11-point smoothing) of CF-CE/CF-UAE (black line), narcissin (blue line), and chlorogenic acid (green line), range 600–1700 cm^−1^ (**b**).

The FTIR spectra of CF-CE and CF-UAE are the same and showed prominent absorption bands at 469, 781, 818, 1030, 1055, 1404, 1456, 1599, 1732, 2855, 2924, and 3318 cm^−1^ (Figure 3a).

A second derivative of the FTIR spectra was utilized to verify the existence of narcissin and chlorogenic acid (primary active compounds) in CF-CE and CF-UAE. To improve the apparent spectral resolution for more precise identification, the Savitzky–Golay polynomial fitting method was implemented (LabSolution IR software, version 1.86 SP2, Shimadzu, Kyoto, Japan) [36,37]. Figure 3b shows the second derivative infrared spectra of CF-CE, CF-UAE, narcissin, and chlorogenic acid.

In the CF-CE and CF-UAE, bands corresponding to narcissin are observed at about 621, 650, 669, 982, 1028, 1124, 1206, 1358, 1601, and 1653 cm^−1^. The bands that can be attributed to the presence of chlorogenic acid are observed at 818, 870, 1057, 1070, 1204, 1516, and 1599 cm^−1^. Based on the DFT analysis, assignments of narcissin and chlorogenic acid bands were proposed (Supplementary Material, Table S2)

**Figure 4:**
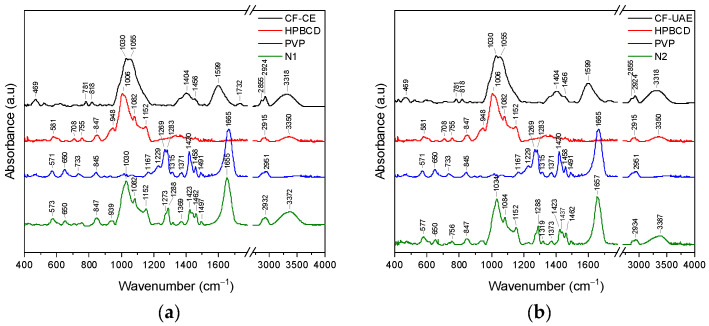

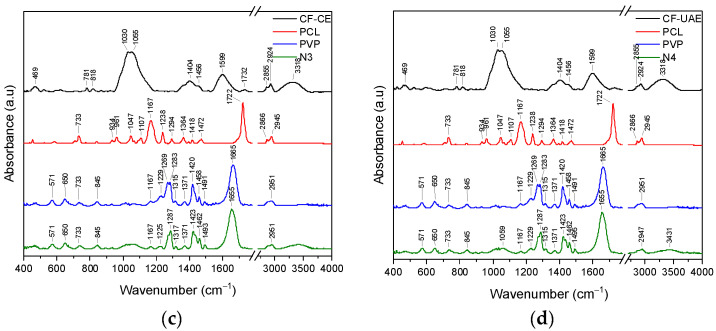
ATR-FTIR analysis of CF-CE (black line), HPβCD (red line), PVP (blue line), and N1 (green line), range 400–4000 cm^−1^ (**a**); CF-UAE (black line), HPβCD (red line), PVP (blue line), and N2 (green line), range 400–4000 cm^−1^ (**b**); CF-CE (black line), PCL (red line), PVP (blue line), and N3 (green line), range 400–4000 cm^−1^ (**c**); CF-UAE (black line), PCL (red line), PVP (blue line), and N4 (green line), range 400–4000 cm^−1^ (**d**).

The ATR-FTIR spectra of CF-CE, CF-UAE, HP-β-CD, PCL, PVP, and nanofibers N1–N4 were compiled to indicate potential interactions between individual components in the nanofibers (N1–N4) (Figure 4).

The ATR-FTIR spectrum of HPβCD showed prominent absorption bands at 847 cm^−1^ (hydrogen bond formation between primary and secondary OH group and the presence of glucopyranose units), 948 cm^−1^ (presence of glucopyranose units), 1006 cm^−1^ (C–H and C–O stretching vibrations), 1082 cm^−1^ (stretching vibration of the C–C and C–O bonds, and wagging vibration of the C–H bonds), 1152 cm^−1^ (C–H and C–O stretching vibrations), 2915 cm^−1^ (C–H stretching of sp3 carbons), and 3350 cm^−1^ (O–H stretching vibrations) [38,39,40,41,42]. The ATR-FTIR spectrum of PVP showed prominent absorption bands at 1167 cm^−1^ (C–C=O), 1229 cm^−1^ (lactone structure), 1283 cm^−1^ (C–N stretching vibrations), 1371 cm^−1^ (–CH deformation vibrations), 1420 cm^−1^ (CH_2_ wagging), 1458 cm^−1^ (CH_2_ bending vibrations), 1665 cm^−1^ (C=O), and 2951 cm^−1^ (C–H stretching vibrations) [42,43,44,45]. The FTIR spectrum of PCL showed prominent absorption bands at 733 cm^−1^ (C–H out-of-plane bending vibration), 1167 cm^−1^ (–C–O–C- symmetric stretching), 1238 cm^−1^ (C–O–C asymmetric stretching), 1294 cm^−1^ (C–O and C–C bands), 1364 cm^−1^ (stretching of OH group), 1472 cm^−1^ (stretching of CH_2_ group), 1722 cm^−1^ (–C=O stretching vibrations of the ester carbonyl group), 2866 cm^−1^ (symmetric stretching of CH_2_ group), and 2945 cm^−1^ (asymmetric stretching of CH_2_ group) [46,47]. A detailed summary of the changes observed in the FTIR spectra for N1 and N3 is summarized in Table S3, while those for N2 and N4 are summarized in Table S4 (Supplementary Materials).

In the spectrum of N1 within the range of ~570–1150 cm^−1^, bands predominantly correspond to HP-β-CD, while in the range of 1270–1700, bands corresponding to PVP were observed. This confirmed the dispersion of CF-CE in the HP-β-CD/PVP matrix. The disappearance of CF-CE bands, and shifts in the characteristic HPβCD bands (571→573 cm^−1^, 948→939 cm^−1^, and 1006→1030 cm^−1^) and PVP bands (1269→1273 cm^−1^, 1283→1288 cm^−1^, 1371→1369 cm^−1^, 1420→1423 cm^−1^, 1458→1462 cm^−1^, 1491→1497 cm^−1^, and 1665→1655 cm^−1^) confirm the interaction between the individual components of the nanofiber. Changes in the position of the HPβCD band observed at about 1006 cm^−1^ suggested that the C–H and C–O groups formed interactions between CF-CE and/or PVP, whereas changes in the position of the PVP band observed at about 1283 cm^−1^ (C–N), 1371 cm^−1^ (–CH), 1420 cm^−1^ (CH_2_), 1458 cm^−1^ (CH_2_), and 1665 cm^−1^ (C=O) suggested that these groups can form interactions with CF-CE and/or HP-β-CD. Analogous changes are observed in the N2 spectrum. The spectra of N3 and N4 have features typical of pure PVP. Bands corresponding to CF-CE/CF-UAE and PCL are not observed. The characteristic bands’ disappearance indicates that CF-CE/CF-UAE and PCL have been fully dispersed in the PVP matrix. Similarly to the case of N1 and N2, here, the shifts in the characteristic PVP bands (1229 cm^−1^ (lactone structure), 1283 cm^−1^ (C–N), 1371 cm^−1^ (–CH), 1420 cm^−1^ (CH_2_), 1458 cm^−1^ (CH_2_), and 1665 cm^−1^ (C=O)) also indicate the participation of these groups in the formation of bonds with CF-CE/CF-UAE and PCL. The PCL/PVP nanofibers showed the distinctive peaks of PVP and PCL nanofibers, proving that the combined spinning of PCL and PVP was successful. Furthermore, no new distinctive peaks were seen in the PCL/PVP nanofibers. This suggests that the PCL and PVP have physical interactions rather than the presence of new chemical bonds in the FTIR spectra of the PCL/PVP nanofibers [31].

**Figure 5:**
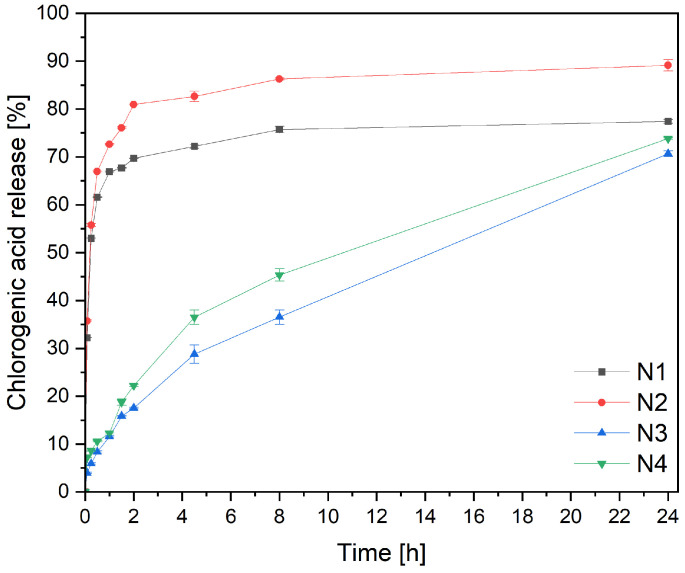
Dissolution profiles of chlorogenic acid from nanofibers N1–N4.

A crucial parameter greatly influences the product’s efficacy: the active ingredient’s release from the nanofibers (Figure 5). For this purpose, a previously developed modified method using baskets was used [27]. A significant difference can be seen in the chlorogenic acid release profiles from PVP/HPβCD-based and PCL/PVP-based nanofibers. In the case of N1 and N2 nanofibers (PVP/HPβCD-based nanofibers), an immediate burst release of chlorogenic acid was observed within the first 30 min. These phenomena can be explained by the benefits of creating nanofibers, which include high load capacity, effective encapsulation, and a high surface area-to-volume ratio, all of which can boost the dissolving rate [48]. Because the cyclodextrin’s high amorphization, wetting, solubilizing, and complexing capabilities increased the solubility of substances, the composition employed to create the nanofibers is also crucial [27,28]. Higuchi kinetics, which postulates that chlorogenic acid is released via diffusion across dispersed vesicles, is the most plausible release mechanism (Table S5, Supplementary Material).

However, in the cases of nanofibers N3 and N4, based on PCL and PVP, prolonged release profiles of chlorogenic acid were observed. Since PCL is a highly hydrophobic polymer and PVP is a highly hydrophilic, the hydrophilicity of PCL/PVP nanofibers can be controlled to provide prolonged substance release by varying the ratio of PCL to PVP in the fibrous matrix [49]. The chlorogenic acid release curves of nanofibers N3 and N4 could be divided into two phases, namely, the burst release phase due to the quick release of amorphous substance present on the surface of the nanofibers, and the final slow-release phase. The second phase exhibits zero-order kinetics characteristics and happens at an exact constant rate of delivery. Korsmeyer–Peppas kinetics with n around 0.4, indicating Fickian diffusion, is the second likely explanation (Table S5, Supplementary Material).

The influence of the type of extract on dissolution behavior within the same matrix was also assessed. For this purpose, the release profiles (N1 and N2, and N3 and N4) were compared using the difference coefficients *f*_1_ and similarity *f*_2_ (Table S6, Supplementary Material). The profiles significantly differed statistically, with larger amounts of chlorogenic acid released from the matrices containing the CF-UAE extract (nanofibers N2 and N4).

The study of antimicrobial activity found that all examined nanofibers are effective at high concentrations of 100 mg/mL or above (Table 4). Nanofiber N4 demonstrated the highest activity with an MIC of 100 mg/mL. Nanofibers N2 and N3 exhibited better efficacy against *Staphylococcus aureus* and *Candida albicans* (MIC 100 mg/mL) than against *Pseudomonas aeruginosa* (MIC 200 mg/mL or above). Nanofiber N1 showed the weakest activity with an MIC of 200 mg/mL or above. The MIC levels for pure chlorogenic acid ranged from 1.25 to 2.5 mg/mL and were compared with the activity of the extract itself [11]. Based on the presented results, the activity of nanofibers against *S. aureus* and *P. aeruginosa* is weaker than what should result from the content of the extract. On the other hand, the activity of nanofibers against *C. albicans* is higher. However, with the entire spectrum of activity, especially against *S. aureus* and *C. albicans* [50,51], one of most important wound pathogens, this does not limit the use of nanofibers.

**Figure 6:**
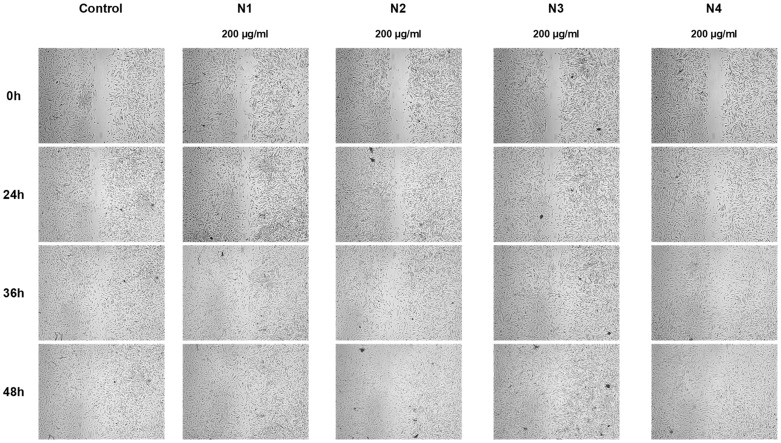
Representative images of wound healing properties of nanofibers N1–N4.

**Figure 7:**
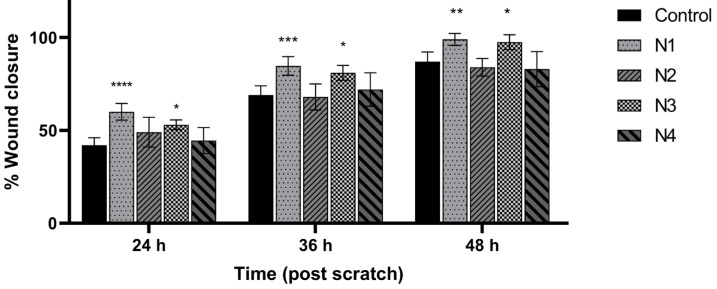
Effect of nanofibers N1–N4 over time on human normal skin fibrocytes’ (Hs27 cells’) ability to close scratches after scratching. The means ± SD of the results are presented. Using a two-way ANOVA and Tukey’s post-hoc test, statistical significance was determined as (*) *p* < 0.05; (**) *p* < 0.01; (***) *p* < 0.001; and (****) *p* < 0.001 (compared to the control at the various time points).

The proposed nanofiber composition meets the requirements for innovative materials for wound treatment, because PVP can store large amounts of water without loss of mechanical integrity and flexible and biocompatible PCL [52]. To observe the wound healing process, a cell line scratch experiment was used. During this process, cells polarize toward the wound and start to protrude, move, and close the wound (Figure 6 and Figure 7). Nanofibers N1 and N3 statistically significantly increased the migration of fibroblasts and the healing of wounds. After 24 and 36 h of incubation, N1 and N3 nanofibers showed even greater wound healing characteristics, with wound closure percentages of up to 61% and 52%, and 85% and 82%, respectively. Using these nanofibers, the wound was 100% closed. Additionally, there was no difference between N1 and N3.

There are no previous reports regarding PVP- and PVP/PCL-based nanofibers containing calendula extract in wound treatment. PCL/calendula-based nanofibers increase fibroblast cell proliferation and attachment [22]. Meanwhile, the healing ability of CS/PEO/CO dressings was assessed in vivo on rat wounds, and 87.5% wound closure was observed after 14 days [19].

**Figure 8:**
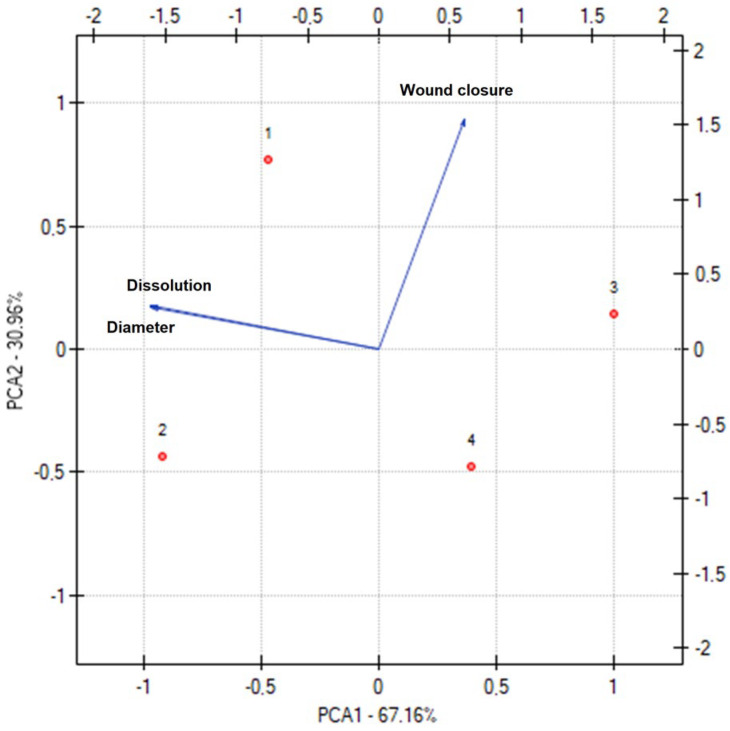
Principal component analysis (PCA) showing the factor loading plot considering the average diameter of nanofibers (=diameter), percentage of chlorogenic acid release at 2 h (=dissolution), and wound closure after 36 h (=wound closure).

Figure 8 shows the results of the PCA analysis for the nanofibers’ characteristics. A statistically significant correlation was indicated between the diameter of the nanofibers and the percentage of released chlorogenic acid (Table S7, Supplementary Material). However, this relationship does not depend directly on the diameter of the nanofibers because the smaller the diameter of the nanofibers, the faster the loaded drug was released from the nanofibers [53]. As described above, the relationship depends on the hydrophilicity and hydrophobicity of the matrix. A very weak negative correlation was noticed between wound closure and drug release behavior, i.e., with the decreasing diameter of the nanofibers, better wound closure was observed, which indicates a greater usefulness of PCL/PVP-based nanofibers.

By observing the dots representing individual nanofibers, two sets can be distinguished in terms of the polymer base (N1 and N2, and N3 and N4) and the extract used (N1 and N3, and N2 and N4). The polymer base significantly impacts both the diameter of the produced nanofibers and the rate of dissolution of active compounds. However, the type of extract was important in examining the wound healing properties. Therefore, the relationships described above are confirmed by the statistical analysis performed.

Based on all the results, structural studies, active substance release kinetics, as well as antibacterial and wound regeneration-improving properties, it was assessed that nanofibers N3 (based on PCL/PVP loaded with CF-CE extract) have the greatest potential in wound treatment.

Notably, the suggested preparation has a high level of clinical utility when considering the outcomes of the experimental investigations. It is important to take note of the patient-friendly form of the nanofibrous mat, which is simple to apply and remove if the dressing needs to be changed, in addition to its proven biological impact on wound healing.

## 4. Conclusions

The nanofibers obtained via electrospinning are characterized by properties that are key to dressings recommended for wound treatment. The scaffolds used fulfilled their function as a structural matrix suitable for releasing the active compounds present in the calendula extract with the required kinetics. Moreover, synergism of action was observed between the biopolymers used and the compounds present in the marigold extract, which are responsible for their healing properties. All nanofibers had highly effective antibacterial properties, promoted fibroblast migration, and accelerated wound healing. Concerning the obtained results, it can be indicated that electrospun nanofibers based on PCL/PVP scaffolds containing CF-CE extract offer the most promising properties in wound treatment.

## Figures and Tables

**Table 1:** Composition of electrospun nanofibers.

	Nanofiber (N1)	Nanofiber (N2)	Nanofiber (N3)	Nanofiber (N4)
CF-CE	0.5 g	-	0.5 g	-
CF-UAE	-	0.5 g	-	0.5 g
PVP	2.0 g	2.0 g	1.9 g	1.9 g
HPβCD	2.0 g	2.0 g	-	-
PCL	-	-	0.5 g	0.5 g
Methanol	10.0 mL	10.0 mL	-	-
Methanol/dichloromethane	-	-	10.0 mL	10.0 mL

PVP—polyvinylpyrrolidone; HPβCD—(2-hydroxypropyl)-β-cyclodextrin; PCL—polycaprolactone.

**Table 2:** Phytochemical and biological characteristics of classical and ultrasound-assisted extracts.

	TPC [mg GAE/1 g Plant Material]	Chlorogenic Acid Content [µg/1 mg of Lyophilized Extract]	Narcissin Content [µg/1 mg of Lyophilized Extract]	Antioxidant Activity IC_50_ [mg/mL]	Anti-Inflammatory Activity IC_50_ [mg/mL]
CF-CE	8.53 ± 1.15 ^a^	9.96 ± 0.24 ^b^	0.26 ± 0.02 ^a^	1.37 ± 0.08 ^a^	10.44 ± 0.45 ^a^
CF-UAE	8.95 ± 1.51 ^a^	10.44 ± 0.08 ^a^	0.27 ± 0.03 ^a^	1.28 ± 0.04 ^a^	9.75 ± 0.50 ^a^

Using Duncan’s test, mean values within a column with the same letter do not differ substantially at *p* < 0.05; the highest values are represented by the first letter of the alphabet, and statistically significant falling values by the following letter.

**Table 3:** Faber distribution and average diameter of nanofibers N1–N4.

	N1	N2	N3	N4
Fiber distribution	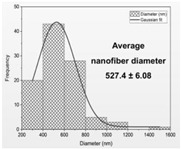	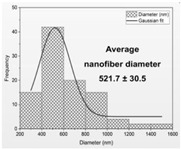	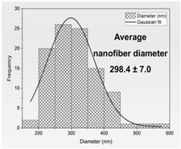	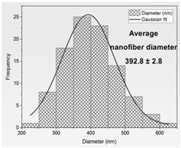
Diameter of nanofibers [nm]	527.40 ± 6.08 ^c^	521.70 ± 30.50 ^c^	298.40 ± 7.00 ^a^	392.80 ± 2.80 ^b^

Using Duncan’s test, mean values within a column with the same letter do not differ substantially at *p* < 0.05; the lowest values are represented by the first letter of the alphabet, and statistically significant falling values by the following letter.

**Table 4:** Minimal inhibitory concentrations (MICs) of nanofibers N1–N4 and chlorogenic acid determined against *Staphylococcus aureus*, *Pseudomonas aeruginosa*, and *Candida albicans*.

Pathogen	MICs (mg/mL)					
	N1	N2	N3	N4	Chlorogenic Acid	Lyophilized Extract [11]
*Staphylococcus aureus*	200	100	100	100	2.5	8
*Pseudomonas aeruginosa*	>200	>200	200	100	2.5	4
*Candida albicans*	200	100	100	100	1.25–2.5	64

## Data Availability

All data supporting reported results can be found within the manuscript and Supplementary Materials.

## Supplementary Materials

The following supporting information can be downloaded at: https://www.mdpi.com/article/10.3390/ma17081736/s1, Table S1. Validation parameters of HPLC method; Table S2. Location and band assignment of narcissin and chlorogenic acid bands observed on the second derivative infrared spectrum of narcissin, chlorogenic acid, and CF-CE/CF-UAE (see Figure 3b). Assignments of narcissin and chlorogenic acid bands made based on DFT calculations with application of 6-31G(d,p) basis set; Table S3. Selected characteristic bonds (in cm^−1^) of CF-CE, HPβCD, PCL, PVP, N1, and N3. Assignments bands of HPβCD [37,38,39,40,41], PVP [41,42,43,44], PCL [45,46] were made based on the literature; Table S4. Selected characteristic bonds (in cm^−1^) of CF-UAE, HPβCD, PCL, PVP, N1, and N3. Assignments bands of HPβCD [37,38,39,40,41], PVP [41,42,43,44], and PCL [45,46] were made based on the literature; Table S5. Parameters of mathematical models fitted to the chlorogenic acid release profiles of nanofibers N1–N4; Table S6. Comparison of the chlorogenic acid release profiles of nanofibers N1–N4; Table S7. Correlation matrix.
