# Supplementary material for: Electrospun Nanofibers Loaded with Marigold Extract Based on PVP/HPβCD and PCL/PVP Scaffolds for Wound Healing Applications
Source: Materials (Basel). 2024 Apr 10;17(8):1736. doi: 10.3390/ma17081736 (PMC11050774; doi:10.3390/ma17081736)
Supplement: Supplementary file 1 [file materials-17-01736-s001.zip › materials-2856673-supplementary.pdf]

materials

MDPI

Supplementary Materials

Electrospun nanofibers loaded with marigold extract based on PVP/HPβCD and PCL/PVP scaffold for wound healing applications

Magdalena Paczkowska-Walendowska [1], Natalia Rosiak [1], Tomasz Plech [2], Tomasz M. Karpiński [3], Andrzej Miklaszewski [4], Katarzyna Witkowska [1], Maciej Jaskólski [1], Cansu Erdem [5] and Judyta Cielecka-Piontek [1,*]

[1] Department of Pharmacognosy and Biomaterials, Poznan University of Medical Sciences, Rokietnicka 3, 60-806 Poznan, Poland; mpaczkowska@ump.edu.pl (M.P.-W.); nrosiak@ump.edu.pl (N.R.); witk.katarzyna@gmail.com (K.W.); jaskolski.mj@gmail.com (M.J.); jpiontek@ump.edu.pl (J.C.-P.)
[2] Department of Pharmacology, Medical University of Lublin, Radziwillowska 11, 20-080 Lublin, Poland; tomasz.plech@umlub.pl (T.P.)
[3] Chair and Department of Medical Microbiology, Medical Faculty, Poznan University of Medical Sciences, Rokietnicka 10, 60-806 Poznan, Poland; tkarpin@ump.edu.pl (T.K.)
[4] Faculty of Mechanical Engineering and Management, Institute of Materials Science and Engineering, Poznan University of Technology, 60-965 Poznan, Poland; andrzej.miklaszewski@put.poznan.pl (A.M.)
[5] Department Pharmaceutical Chemistry, Ege Üniversitesi, 35040 İzmir, Turkey; cansueerdem@gmail.com (C.E.)

* Correspondence: jpiontek@ump.edu.pl

Table S1. Validation parameters of HPLC method

Parameter	**Chlorogenic acid**	**Narcissin**
Linearity: y = ax + b		
$a \pm S_a$	0.3560 ± 0.0045	0.6066 ± 0.0059
$b \pm S_b$	insignificant (α=0.05)	insignificant (α=0.05)
Correlation coefficient (r)	0.9999	0.9999
Range of linearity [µg/mL]	130.00–520.00	140.00–550.00
Intra-day precision, RSD (<5% required) = repeatability		
The lowest concentration	0.18	3.39
The middle concentration	0.84	0.36
The lowest concentration	1.35	4.15
Limit of detection (LOD) [µg/mL]	12.06	8.24
Limit of quantification (LOQ) [µg/mL]	36.53	24.96

Table S2. Location and band assignment of narcissin and chlorogenic acid bands observed on the second derivative infrared spectrum of narcissin, chlorogenic acid and CF-CE/ CF-UAE (see Figure 3b). Assignments of of narcissin and chlorogenic acid bands made based on DFT calculations with application of 6-31G(d,p) basis set

**CF-CE/ CF-UAE [cm^{-1}]**	**narcissin [cm^{-1}]**	**chlorogenic acid [cm^{-1}]**	**Assignments**

621	621		O-H wagging
650	650		O-H wagging
669	669		O-H wagging
818		818	C-C-C assymetric stretching + C-H twisting
868		870	CH_2 twisting + C-O-H rocking
980	982		C-C-C symetric stretching + C-O-H rocking
1028	1028		C-O-H rocking
1059		1057	C-C-C assymetric stretching + CH_2 twisting + C-H scissoring
1078		1070	C-C-C assymetric stretching + CH_2 twisting + C-H scissoring
1124	1124		C-H wagging
1206	1206		C-H wagging and scissoring + C-O-H scissoring
		1204	CH_2 twisting + C-H wagging + O-H scissoring
1358	1358		C-O-H scissoring + C-C-C scissoring + C-H scissoring
1514		1516	C-O-H scissoring + C-C-C assymetric stretching + C-H rocking
1601	1601		C-O-H scissoring + C-O-C scissoring + C-C-C assymetric stretching and scissoring
		1599	C-C-C scissoring + C-O-H scissoring + C=C-C assymetric stretching
1653	1653		C-C-C assymetric stretching + C-O-H scissoring + C-H rocking

Table S3. Selected characteristic bonds (in cm^{-1}) of CF-CE, HPβCD, PCL, PVP, N1, and N3. Assignments bands of HPβCD [38–42], PVP [42–45], PCL [46,47] were made based on literature

CF-CE [cm^{-1}]	HPβCD [cm^{-1}]	PCL [cm^{-1}]	PVP [cm^{-1}]	N1 [cm^{-1}]	N3 [cm^{-1}]	Assignments
469				none	none	
			571	573	571	
		733			↓ intensity	C–H out of plane bending vibration
781				none	none	
818				none	none	chlorogenic acid: C-C-C assymetric stretching + C-H twisting
			845	none	845	
	847			847		hydrogen bond formation between primary and secondary OH group and the presence of glucopyranose units
		934			↓ intensity	
	948			939		presence of glucopyranose units
		961			↓ intensity	
	1006			1030		C–H, C–O stretching vibrations
1030				none	none	narcissin: C-O-H rocking
		1047			none	
1055				none	none	chlorogenic acid: C-C-C assymetric stretching + CH_2 twisting + C-H scissoring
	1082			1082		stretching vibration of the C–C, C–O bonds, and wagging vibration of the C–H bonds
		1107			none	
	1152			1152		C–H, C–O stretching vibrations

		1167			none	–C–O–C- symmetric stretching
			1167	none	1167	C–C=O
			1229	none	1225	lactone structure
		1238			none	C–O–C asymmetric stretching
			1269	1273	↓ intensity	
			1283	1288	1287	C–N stretching vibrations
		1294			none	C–O and C–C bands
		1364			none	stretching of OH group
			1371	1369	1371	–CH deformation vibrations
1404				none	none	
		1418			none	
			1420	1423	1423	CH_2 wagging
1456				none	none	
			1458	1462	1462	CH_2 bending vibrations
		1472			none	stretching of CH_2 group
			1491	1497	1493	
1599				none	none	chlorogenic acid: C-C-C scissoring + C-O-H scissoring + C=C-C assymetric stretching
			1665	1655	1655	C=O
		1722			none	–C=O stretching vibrations of the ester carbonyl group
1732				none	none	
2855				none	none	
		2866			↓ intensity	symmetric stretching of CH_2 group
	2915			none		C–H stretching of sp3 carbons
2924				none	none	
		2945			none	asymmetric stretching of CH_2 group
			2951		2951	C–H stretching vibrations
3318				3372	3404	
	3350			3372		O–H stretching vibrations

Table S4. Selected characteristic bonds (in cm^{-1}) of CF-UAE, HPβCD, PCL, PVP, N1, and N3. Assignments bands of HPβCD [38–42], PVP [42–45], PCL [46,47] were made based on literature

CF-UAE [cm^{-1}]	HPβCD [cm^{-1}]	PCL [cm^{-1}]	PVP [cm^{-1}]	N2 [cm^{-1}]	N4 [cm^{-1}]	Assignments
469				none	none	
			571	577	571	
		733			none	C–H out of plane bending vibration
781				none	none	
818				none	none	chlorogenic acid: C-C-C assymetric stretching + C-H twisting
			845	none	845	
	847			847		hydrogen bond formation between primary and secondary OH group and the presence of glucopyranose units
		934			none	
	948			↓ intensity		presence of glucopyranose units
		961			none	
	1006			1034		C–H, C–O stretching vibrations
1030				none	none	narcissin: C-O-H rocking
		1047				
1055				none	1059	chlorogenic acid: C-C-C assymetric stretching + CH_2 twisting + C-H scissoring
	1082			1084		stretching vibration of the C–C, C–O bonds, and wagging vibration of the C–H bonds
		1107			none	
	1152			1152		C–H, C–O stretching vibrations
		1167			none	–C–O–C- symmetric stretching
			1167	none	1167	C–C=O
			1229	none	1229	lactone structure
		1238			none	C–O–C asymmetric stretching
			1269	↓ intensity	↓ intensity	
			1283	1288	1287	C–N stretching vibrations
		1294			none	C–O and C–C bands
		1364			none	stretching of OH group
			1371	1373	1371	–CH deformation vibrations
1404				none	none	
		1418			none	
			1420	1423	1423	CH_2 wagging
1456				none	none	
			1458	1462	1462	CH_2 bending vibrations
		1472			none	stretching of CH_2 group
1599				none	none	chlorogenic acid: C-C-C scissoring + C-O-H scissoring + C=C-C assymetric stretching
			1665	1657	1655	C=O
		1722			none	–C=O stretching vibrations of the ester carbonyl group

2855				none	↓ intensity	
		2866			none	symmetric stretching of CH_2 group
	2915			none		C–H stretching of sp3 carbons
2924				none	none	
		2945			2947	asymmetric stretching of CH_2 group
			2951	2934	none	C–H stretching vibrations
3318				3387	3431	
	3350			3387		O–H stretching vibrations

Table S5. Parameters of mathematical models fitted to the chlorogenic acid release profiles of nanofibers N1-N4

Formulation	Mathematical model							
No.	**Zero-order kinetic**		**First-order kinetic**		**Higuchi kinetic**		**Korsmeyer-Peppas kinetic**	
	K	R^2	K	R^2	K	R^2	R^2	n
N1	1.49	0.21	0.05	0.08	**7.72**	**0.79**	0.63	0.53
N2	1.82	0.24	0.05	0.09	**9.00**	**0.83**	0.58	0.40
N3	**2.76**	**0.94**	0.64	0.30	5.50	0.76	**0.95**	**0.40**
N4	**2.86**	**0.87**	0.11	0.44	6.13	0.82	**0.90**	**0.40**

the most fitting mathematical model is shown in bold

Table S6. Comparison of the chlorogenic acid release profiles of nanofibers N1-N4

	N1	N2	N3
N1			
N2	f_1 = 12.11<br>f_2 = 32.79		
N3	f_1 = 65.44<br>f_2 = 3.82	f_1 = 69.17<br>f_2 = 7.51	
N4	f_1 = 59.15<br>f_2 = 1.63	f_1 = 63.57<br>f_2 = 5.68	f_1 = 18.17<br>f_2 = 46.98

Table S7. Correlation matrix

	Diameter	Dissolution in 2 hours	Wound closure in 36 hours
Diameter		0.9433	-0.1924
Dissolution in 2 hours	0.9433		-0.1880
Wound closure in 36 hours	-0.1924	-0.1880	
